# Clinical effectiveness of usual care with or without antidepressant medication for primary care patients with minor or mild-major depression: a randomized equivalence trial

**DOI:** 10.1186/1741-7015-5-36

**Published:** 2007-12-07

**Authors:** Marleen LM Hermens, Hein PJ van Hout, Berend Terluin, Herman J Adèr, Brenda WJH Penninx, Harm WJ van Marwijk, Judith E Bosmans, Richard van Dyck, Marten de Haan

**Affiliations:** 1 Department of General Practice, Institute for Research in Extramural Medicine, VU University Medical Center, Van der Boechorststraat 7, 1081 BT Amsterdam, The Netherlands; 2 Department of Clinical Epidemiology and Biostatistics, Institute for Research in Extramural Medicine, VU University Medical Center, Van der Boechorststraat 7, 1081 BT Amsterdam, The Netherlands; 3 Department of Psychiatry, VU University Medical Center/GGZ Buitenamstel and Institute for Research in Extramural Medicine, Valeriusplein 9, 1075 BG Amsterdam, The Netherlands; 4 Health Technology Assessment Unit, Institute for Research in Extramural Medicine, VU University Medical Center, Van der Boechorststraat 7, 1081 BT Amsterdam, The Netherlands

## Abstract

**Background:**

Minor and mild-major depression are highly prevalent in primary care. There is insufficient evidence for the effectiveness of antidepressants in the treatment of minor and mild-major depression. We compared the effectiveness of usual primary care treatment, with or without antidepressants, in minor and mild-major depression.

**Methods:**

A pragmatic patient-randomized equivalence trial with 52 weeks follow-up was conducted in The Netherlands. In total, 59 primary care physicians (PCPs) recruited and treated 181 adult patients with minor or mild-major depression. Patients were randomized to four consultations within 3 months of usual care plus antidepressants (UCandAD) or usual care alone (UCnoAD). The Montgomery Åsberg Depression Rating Scale (MADRS) was used to assess changes in severity of depressive symptoms. The predefined equivalence margin was set at five points. Multilevel analysis was used to analyze the data. Secondary outcome measures were the Short-Form 36 (SF-36), and the Client Satisfaction Questionnaire (CSQ-8).

**Results:**

Patients received on average 3.0 (SD 1.4) 15-min consultations within 3 months with (n = 85) or without paroxetine (n = 96). Equivalence of UCandAD and UCnoAD was demonstrated in the intention-to-treat analyses as well as the per-protocol analysis after 6 weeks, but not at 13, 26 and 52 weeks follow-up. No statistical differences in effectiveness between treatment groups were found in the intention-to-treat analysis. No differences in the physical and mental functioning (SF-36) were found between the treatment groups. Patients allocated to UCandAD were slightly more satisfied with their treatment at 13 weeks follow-up (but not at 52 weeks follow-up) than patients allocated to UCnoAD. Preliminary analyses suggested that subgroups such as patients with mild-major (instead of a minor) depression might benefit from antidepressant treatment. Patients who were assigned to their preferred treatment (in particular to UCnoAD) were more often compliant and had better clinical outcomes.

**Conclusion:**

UCandAD was as effective as UCnoAD over the first 6 weeks, but not at 13, 26, and 52 weeks. However, superiority of either treatment could not be demonstrated either. The question whether antidepressants add any clinical effect to usual care remains unresolved. We recommend future studies to look for subgroups of patients who may benefit from antidepressants.

**Trial registration:**

Dutch Trial Registry ISRCN03007807.

## Background

In primary care, minor and mild-major depressions are more common than severe major depressive disorders [1]. The estimated point prevalence of minor depression among primary care patients varies between 3.4% and 4.7% [1]. There is insufficient evidence for the effectiveness of antidepressants in the treatment of minor and mild-major depression in primary care [2-7]. Potential hazards of antidepressants are stigmatization, medicalization, dependence, and undesirable side effects. In general, antidepressants are only recommended when depressive symptoms are persistent and patients experience severe functional impairment [2,7-9]. In minor depression, specific treatments may not be indicated as there are high rates of improvement with watchful waiting [10].

It is interesting that, from an international perspective, the same body of scientific evidence on the treatment of minor and mild major depression has been interpreted differently. British guidelines favor some restraint in the prescription of antidepressants [5,6], while the American guidelines favor the use of these medications [11]. Dutch depression guidelines, like the British guidelines, recommend refraining from the prescription of antidepressants in patients with minor or mild-major depression [12]. However, the prescription rate of antidepressants increases year by year [13] and it seems that a substantial portion of the antidepressants is prescribed to this group [8,9]. In our opinion, prescription of antidepressants in patients with minor or mild-major depression would only be justified if antidepressants are shown to have additional benefits over non-pharmacological care alone.

The primary aim of the present study was to examine whether antidepressant medication (UCandAD) adds any clinical effectiveness to non-pharmacological usual care (UCnoAD) by the primary care physician (PCP) in patients with minor or mild-major depression. As we hypothesized that antidepressants would have no additional effects in comparison with usual care, we designed an equivalence trial.

## Methods

### Design

We conducted a randomized controlled trial in which adult primary care patients with minor or mild-major depression were randomized to receive either usual care plus 3 months of antidepressant treatment (UCandAD) or usual care alone (UCnoAD). Because we were interested in the treatment effectiveness in everyday practice, we decided to conduct a pragmatic trial, implying that the interventions were provided by PCPs to typical primary care patients under normal practice circumstances. UCandAD was our treatment of interest, the experimental treatment. We did not want to compare antidepressant medication with placebo medication as such a treatment is not a feasible alternative to medication in daily practice. Instead, our control intervention, usual care, was based on the guideline on depression issued by the Dutch College of General Practitioners [12]. We hypothesized that UCandAD was as effective as, i.e. equivalent to, UCnoAD. Therefore, we designed an equivalence trial. The investigation was carried out in accordance with the latest version of the Declaration of Helsinki [14]. The Medical Ethical Committee of the VU University Medical Center approved the study design.

### Participants

The study was conducted in 2002 and 2003. PCPs in the west and middle of The Netherlands were invited to participate in this trial. During a practice visit the PCPs were informed about the study's aim and procedures. Participating PCPs recruited, diagnosed, and treated consecutive eligible patients themselves. Patients were considered eligible if they had been diagnosed by their PCP as suffering from a current episode of minor or mild-major depression (i.e. three to six out of nine DSM-IV symptoms of depression, including at least one of the core symptoms, 'sadness' or 'loss of pleasure'). In accordance with the Dutch guideline on depression we defined minor depression as a depressive disorder with three to four DSM-IV depressive symptoms [12]. Largely in accordance with the Diagnostic and Statistical Manual of Mental Disorders, 4th edition (DSM-IV [15]), we defined mild-major depression as a depressive disorder with five to six symptoms. The symptoms had to be present nearly every day for at least 2 weeks. Also, they had to cause clinically significant distress or impairment in social, occupational or other important areas of functioning.

Patients were excluded from the trial for the following reasons: age under 18 years, currently on antidepressant medication, currently receiving psychological therapy, experiencing psychotic features, addiction to alcohol or drugs, loss of a loved one or significant other in the past 6 months, pregnancy or breastfeeding, inability to complete questionnaires because of language difficulties, illiteracy or cognitive decline, or not having a telephone.

When the PCP considered a patient to be eligible for the study, a research assistant made an appointment for a baseline interview at the patient's home. At the start of the interview, the patient received a full explanation of the study and written informed consent was obtained.

### Randomization

Patients were randomly assigned to one of the treatment conditions. Block randomization (block size 4) was used to ensure equal numbers of patients in the two conditions per PCP. Allocation schemes were generated by random number tables before the trial started. After the baseline interview with the patient, staff not in contact with the patient opened the appropriate opaque sealed envelope. PCPs and patients were not informed about the allocated treatment until the first treatment session. Due to the nature of the study, blinding of patients, research assistants or PCPs was not possible.

### Interventions

Before the start of the trial, PCPs received a 3-h training session to improve recognition of patients with minor and mild-major depression, to make a standardized DSM-IV depression diagnosis, and to refresh their treatment skills.

All patients were scheduled for four 10–20 min consultations with the PCP at 2, 4, 7, and 11 weeks after inclusion. During these consultations, patients randomized to the usual care condition (UCnoAD) received patient education, information about depression and its prognosis, and advice on how to deal with depression (focus on the present, maintain social activities and daily routines, exercise, putting achievable goals, and restrain alcohol usage). This treatment may be considered as a standardized form of usual PCP care. Patients in this condition were not to receive any antidepressant medication.

Patients randomized to the antidepressant condition (UCandAD) received the selective serotonin reuptake inhibitor (SSRI) paroxetine beside the usual care treatment described above. The paroxetine dose was 20 mg/day for at least 3 months, along with education about the effects and side-effects of paroxetine. After 4 weeks the dose could be increased to 40 mg/day in case of poor clinical response. In case of intolerance to paroxetine, sertraline was advised. Paroxetine was chosen as first choice antidepressant because it was the most commonly prescribed antidepressant drug in The Netherlands at the time the study started [13], and no clinically meaningful differences between antidepressants are found in primary care patients [16].

In both conditions, prescription of benzodiazepines was allowed in patients with severe sleeping disturbances. After 3 months, treatment could end or continue in the way PCPs and patients preferred.

In equivalence trials, it is important to optimize the contrast between the two treatment conditions to assure a conservative approach. Therefore, we gave PCPs some instructions on how to shape both treatments in such a way that the two interventions would differ mostly with respect to the use of antidepressants. During the first 3 months, PCPs were asked to give patients in both conditions the same number of consultations and not to deviate from the medication protocol unless the PCP judged this to be imperative. To prevent contamination from co-interventions, PCPs were asked to refrain from referral to specialized mental health care (e.g. to a psychiatrist, psychologist, psychotherapist, or a social worker).

### Assessments

Sociodemographic and clinical information was collected at baseline, comprising the List of Threatening Experiences Questionnaire (LTE-Q [17]), neuroticism (a subscale of the NEO-FFI [18]), duration and history of depressive symptoms, and chronic somatic diseases. As a check of the diagnoses of the PCP, but without consequences for the inclusion in the study, standardized psychiatric diagnoses according to DSM-IV criteria were obtained with the Composite International Diagnostic Interview (CIDI [19]). In addition, we collected both patients' and PCPs' treatment preferences before randomization.

### Primary outcome measure

The Montgomery Åsberg Depression Rating Scale (MADRS [20], observer rating scale, 10 items, scale range 0–60, higher scores indicating more severe depressive symptoms) was used to assess changes in severity of depressive symptoms over 52 weeks of follow-up. The MADRS was administered face-to-face at baseline, and by telephone at 6, 13, 26, and 52 weeks follow-up by the same research assistant. No differences in the mode of administration of the MADRS (i.e. by telephone versus face-to-face) were found [21].

### Secondary outcome measure

The Short-Form 36 (SF-36 [22,23]) was completed by patients during the baseline interview and was sent to the patients' home at 6, 13, and 52 weeks follow-up. The SF-36 is a self-report quality of life measure, consisting of eight scales that are aggregated into two summary measures: the Physical (PCS) and Mental (MCS) Component Summary scores. Lower scores indicate worse health, higher scores indicate better health.

The Client Satisfaction Questionnaire (CSQ-8 [24], self-report scale, eight items, item range 1–4, higher scores indicating higher satisfaction with the treatment received) was sent to the patients' home at 13 and 52 weeks follow-up.

Regarding the secondary outcomes we were only interested in possible differences between treatment groups, and thus not in demonstrating equivalence.

### Equivalence margins

The equivalence margin for improvement in MADRS score was set at five points. A five-point difference in MADRS score corresponds to 0.5 standard deviation of the MADRS change score (mean 12.5; SD 10.0) at 6 months follow-up in a comparable primary care study in patients allocated to placebo treatment [25]. Half a standard deviation is considered to represent a clinically relevant effect [26]. Our main hypothesis in this clinical evaluation was that UCandAD by the PCP was as effective (i.e. equivalent) in reducing depressive symptoms as UCnoAD. This hypothesis was accepted if the 95% confidence interval (CI) of the difference in improvement in MADRS scores between UCandAD and UCnoAD at different time points was between -5 and 5.

### Statistical analysis

Using the sample size calculation of Jones et al for equivalence trials [27], based on 90% power (1-β) to detect a clinically relevant difference in improvement of -5 points on the MADRS (α = 0.05, two-sided), 84 patients were required in each group.

To estimate the CIs for the mean difference between the groups, we used parameter estimates of repeated measures multilevel analysis in MLwiN [28]. This method is characterized by an unrestricted repeated measurements design, allowing all observations to be used. Multilevel analysis was used for both the intention-to-treat analyses and the per-protocol analyses. To study change in MADRS scores, baseline measurements were used as a covariate as the improvement in depressive symptoms may be different for patients with higher versus lower MADRS baseline scores. In all analyses, all available time points were analyzed simultaneously.

Additionally, an adjusted model was analyzed. We adjusted for additional specialized help from mental health services during the experimental period, which was considered a co-intervention, to check the robustness of our outcomes.

In equivalence trials it is recommended to perform a per-protocol analysis in all cases; intention-to-treat analysis is no longer considered conservative [27,29]. When equivalence is demonstrated in both the intention-to-treat and the per-protocol analysis, the evidence is considered to be strongest. We performed both intention-to-treat and per-protocol analysis. If equivalence of the two treatments could not be established, we explored superiority of either treatment. As intention-to-treat analyses has been recognized as the most conservative strategy for superiority trials to analyse data, we focused at the intention-to-treat analysis. First statistical significance was explored (95% CI < 0 or 95% CI > 0), then clinical superiority was explored (95% CI < -5 or 95% CI > 5).

In the intention-to-treat analyses all patients were analyzed according to group assignment. Per-protocol analyses were performed for patients who received at least 70 defined daily doses (DDDs) of an antidepressant drug in the first 3 months when in the UCandAD group, and patients who received no more than 30 DDDs of an antidepressant drug in the first 3 months when in the UCnoAD group. Thus, protocol violation has a different meaning in the treatment groups; violators in the UCandAD group received a less extensive treatment (no antidepressants), whereas violators in the UCnoAD group received a more intensified treatment (additional antidepressants).

Differences in protocol violation between treatment groups were analyzed using the chi-square test (SPSS 11.0). To find predictors of protocol violation logistic regression was used for each treatment group separately. The influence of gender, age, the presence of life events, duration and history of depressive symptoms, chronic diseases, neuroticism, severity of depression diagnosis at baseline (i.e. minor versus mild-major depression, as assessed by the PCP), and use of mental health services during the first 3 months were explored.

In addition, some explorative analyses were performed. Differences in benzodiazepines prescription between treatment groups and differences in protocol violation in combination with patient's treatment preferences and assignment were analyzed using the chi-square test in SPSS 11.0 (SPSS Inc., Chicago, Il).

The secondary outcomes, the SF-36 and the CSQ-8, were explored for statistical significant differences in the intention-to-treat analyses. We used multilevel analysis in MLwiN [28] for the SF-36. For the CSQ-8, total mean item scores were computed and the t statistic in SPSS 11.0 was used (two-sided, p < 0.05).

## Results

### Patients

**Figure 1:**
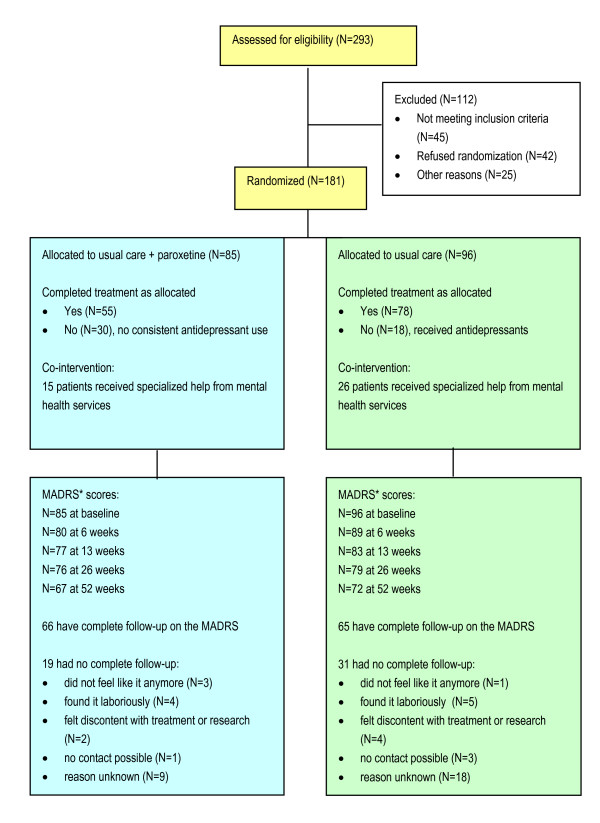
**Flow chart of patients through trial**. MADRS, Montgomery Åsberg Depression Rating Scale.

Patients were recruited from February 2002 to March 2003. Of 117 participating PCPs, 59 assessed 293 contacting patients for eligibility. As a result, 181 patients were randomly assigned to one of the two treatments (Figure 1). No differences in baseline sociodemographic and clinical characteristics between the patients in the two treatment conditions (Table 1) were found.

**Table 1:** Baseline demographic, socioeconomic, and clinical characteristics of participants of each group. Values are numbers (percentages) of patients unless stated otherwise.

Baseline characteristic	Usual care + paroxetine (n = 85)	Usual care (n = 96)
Age (mean (SD))	46 (16)	46 (16)
Women	62 (73%)	70 (73%)
Minor depression diagnosis PCP*	15 (18%)	16 (17%)
Major Depressive Disorder (CIDI)†	56 (68%)	59 (65%)
Mild	21 (26%)	15 (16%)
Moderate	17 (21%)	25 (27%)
Severe	18 (22%)	19 (21%)
Dysthymia (CIDI)†	14 (17%)	17 (19%)
Baseline MADRS‡ (Mean (SD))	23.7 (10.4)	24.1 (10.7)
Private insurance	19 (22%)	20 (21%)
Dutch ethnic group	72 (85%)	80 (83%)
Partner	61 (72%)	58 (60%)
Higher education§	20 (24%)	24 (25%)
Employed	53 (62%)	61 (64%)
Mental health care use	3 (3.1%)	2 (2.4%)
Neurotic (high)¶	40 (47%)	48 (50%)
Chronic disease (≥ 1)	33 (39%)	31 (32%)
Life events (≥ 1)#	63 (74%)	69 (72%)
Duration depression > 3 months	52 (61%)	61 (64%)
History of depression	64 (75%)	78 (81%)
Treatment history of depression	38 (45%)	45 (47%)
Patient's treatment preference:		
Usual care + paroxetine	16 (19%)	19 (20%)
Usual care	30 (35%)	39 (41%)
No preference	39 (46%)	38 (40%)
Physician's treatment preference:		
Usual care + paroxetine	26 (31%)	22 (23%)
Usual care	6 (7%)	14 (15%)
No preference	32 (38%)	43 (45%)
No preference registered	21 (25%)	17 (18%)

*Three to four depressive symptoms correspond to minor depression, five to six to mild-major depression.

†Composite International Diagnostic Interview (CIDI) diagnosis of 173 patients were obtained (96%); 82 patients allocated to usual care + paroxetine, 91 patients allocated to usual care.

‡Montgomery Åsberg Depression Rating Scale.

§At least 11 years of education.

¶As measured with the Neuroticism Scale of the NEO-FFI. A scale score of ≥ 40 was considered high, < 40 as low (range 12–60, median was 40).

#As measured with a translated and, with permission of the author, adapted version of the List of Threatening Experiences Questionnaire (LTE-Q).

For 160 patients, self-report information and/or information from the pharmacist on antidepressants during the first 13 weeks could be used to determine whether patients had or had not violated the treatment protocol. For 21 patients the necessary information was incomplete or missing and we contacted the PCP for information on antidepressant prescription in order to decide whether protocol violations had occurred.

At baseline, 43% of the patients had no preference for any of the two treatments (77/181); 19% preferred UCandAD (35/181), and 38% preferred UCnoAD (69/181). PCPs registered their treatment preference at baseline in 79% of the patients (143/181). In 52% of the cases the PCP did not have any preference regarding the treatment (75/143); in 34% of the cases the PCP preferred UCandAD (48/143), and in 14% the PCP preferred UCnoAD (20/143).

### Interventions

The mean number of consultations with the PCP in the first 3 months, ascribable to our study, was 3.0 (SD 1.4), and each lasted on average 15.1 min (SD 0.7) with no differences between UCandAD (mean 3.1; SD 1.3) and UCnoAD (mean 2.9; SD 1.4). During these consultations, the PCP evaluated the depressive symptoms (82%), discussed the course of the depressive symptoms (76%), and talked with the patient about their functioning at home or at work (80%). Slightly less attention was paid to patient education on depression (58%), emotional support (71%), giving practical advice (64%), and formulating achievable goals (55%). In patients receiving UCandAD, the PCP also paid attention to the effects and side-effects of antidepressants (62%), the prognosis as a result of antidepressant use (54%), and the importance of compliance with the antidepressant therapy (57%). A total of 41 patients (23% of 181 patients) received additional specialized help from mental health services during the experimental period; 15 in the UCandAD group and 26 in the UCnoAD group (p > 0.05). For 127 patients (70% of 181), information on the prescription of benzodiazepines was available. A total of 43 patients received additional benzodiazepines; 19 in the UCandAD group and 24 in the UCnoAD group (p > 0.05).

### Primary outcome: MADRS

**Figure 2:**
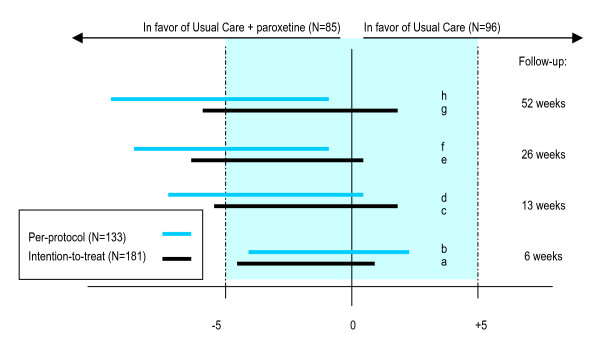
**Differences between treatment groups in Montgomery Åsberg Depression Rating Scale (MADRS) scores, in relation to equivalence**. Schematic presentation. Horizontal bars indicate two-sided 95% confidence intervals (CIs). Blue tinted area indicates zone of equivalence. Bars a and b: The 95% CI of the difference in symptom change lies between the equivalence margins of -5 and 5 points difference; equivalence of both treatments is demonstrated. Bars f and h: The 95% CI of the difference in symptom change lies entirely to the left of zero; a statistical significant difference in favor of usual care + paroxetine is demonstrated.

Table 2 presents the change in the severity of the depressive symptoms and 95% CIs for both treatment groups over 52 weeks. The results of Table 2 are visualized in Figure 2. As randomization had been successful (Table 1) we did not explore potential effect modifiers or confounders. Table 3 shows that additional specialized help from mental health services during the first 3 months biased the results to no difference (e.g. adjusted 95% CI at 6 weeks -5.5 to 2.1 versus unadjusted 95% CI at 6 weeks -4.5 to 2.4 in the per-protocol analyses).

**Table 2:** Main outcome table. Baseline Montgomery Åsberg Depression Rating Scale (MADRS) scores and change in the depressive symptoms over 52 weeks for patients in the intention-to-treat and per-protocol analysis. Values are means unless stated otherwise. Estimated mean differences and 95% Confidence Intervals (CIs) are presented.

	Intention-to-treat MADRS (n = 181)				
	Usual care + paroxetine (n = 85)	Usual care (n = 96)	Mean difference*	SD	95% CI

Baseline score	23.7	24.1			
6 weeks – baseline†	-7.6	-6.0	-1.6	20.2	-4.7; 1.4‡
13 weeks – baseline †	-10.2	-8.7	-1.5	22.5	-5.1; 1.9
26 weeks – baseline †	-13.0	-10.0	-3.0	21.3	-6.4; 0.3
52 weeks – baseline †	-14.7	-12.6	-2.1	24.1	-6.1; 1.9

	Per-protocol MADRS (n = 133)				
	
	Usual care + paroxetine (n = 55)	Usual care (n = 78)	Mean difference*	SD	95% CI

Baseline score	25.1	24.1			
6 weeks-baseline†	-7.8	-6.7	-1.1	20.0	-4.5; 2.4‡
13 weeks-baseline†	-12.1	-9.0	-3.1	20.7	-6.8; 0.5
26 weeks-baseline†	-13.9	-9.3	-4.6	20.6	-8.4; -0.9§
52 weeks-baseline†	-17.6	-12.6	-5.0	21.2	-9.1; -1.0§

*A negative mean difference is a difference in favor of usual care + paroxetine.

†A negative score means a decrease in the severity of symptoms.

‡The 95% CI of the difference in symptom change lies entirely between the equivalence margins of -5 and +5 points, indicating equivalence of usual care + paroxetine and usual care.

§The 95% CI of the difference in symptom change lies entirely to the left of zero, indicating statistical significant differences in favor of usual care + paroxetine.

**Table 3:** Baseline Montgomery Åsberg Depression Rating Scale (MADRS) scores and change in the depressive symptoms over 52 weeks for patients in the intention-to-treat and per-protocol analysis. Results are adjusted for additional specialized help from mental health services during the first 3 months. Values are means unless stated otherwise. Estimated mean differences and 95% CIs are presented.

	Intention-to-treat MADRS (n = 181)				
	
	Usual care + paroxetine (n = 85)	Usual care (n = 96)	Mean difference*	SD	95% CI
Baseline score	23.8	24.2			
6 weeks – baseline†	-7.7	-5.8	-1.9	20.7	-5.0; 1.2
13 weeks – baseline †	-10.9	-8.4	-2.5	23.4	-6.1; 1.1
26 weeks – baseline †	-13.1	-10.1	-3.0	21.4	-6.4; 0.3
52 weeks – baseline †	-14.8	-12.7	-2.1	24.2	-6.2; 1.9

	Per-protocol MADRS (n = 133)				
	
	Usual care + paroxetine (n = 55)	Usual care (n = 78)	Mean difference*	SD	95% CI

Baseline score	25.2	24.1			
6 weeks-baseline†	-8.2	-6.5	-1.7	22.1	-5.5; 2.1
13 weeks-baseline†	-13.3	-8.7	-4.6	24.3	-8.9; -0.3‡
26 weeks-baseline†	-14.0	-9.3	-4.8	20.6	-8.5; -1.0‡
52 weeks-baseline†	-17.7	-12.5	-5.2	21.2	-9.2; -1.2‡

*A negative mean difference is a difference in favor of usual care + paroxetine.

†A negative score means a decrease in the severity of symptoms.

‡The 95% CI of the difference in symptom change lies entirely to the left of zero, indicating statistical significant differences in favor of usual care + paroxetine.

### Findings

Table 2 shows that, in the intention-to-treat (n = 181) as well as the per-protocol analysis (n = 133), equivalence of UCandAD and UCnoAD could be demonstrated at 6 weeks follow-up (the 95% CI of the difference in improvement in MADRS scores between the treatment groups lies between the equivalence margins of -5 and 5). However, at 13, 26, or 52 weeks follow-up equivalence could not be demonstrated.

Superiority of either treatment could not be demonstrated; no statistical significant difference in effectiveness of either treatment could be demonstrated at any time point in the intention-to-treat analysis.

### Protocol violations

Protocol violations more often occurred in UCandAD (30 of 85 patients) than in UCnoAD (18 of 96 patients) (p < 0.02). Specialized help from mental health services at 13 weeks (p < 0.01) predicted protocol violations in UCandAD. Chronic disease (p = 0.04) and specialized help from mental health services at baseline (p < 0.02) were predictors of protocol violation in UCnoAD. Explorative analyses showed that patients who preferred UCnoAD violated the protocol more often when assigned to UCandAD than when assigned to UCnoAD (number of violations 11/30 vs 5/39, p = 0.02). Patients who had no treatment preference had comparable results (number of violations 16/39 vs 5/38, p < 0.01). Patients who preferred UCandAD violated the protocol more often when assigned to UCnoAD than when assigned to UCandAD (number of violations 8/19 vs 3/16, p value not significant).

To get an impression of the effect of protocol adherence on the MADRS scores, the intention-to-treat analyses were compared with the per-protocol analyses. The only patients for whom marked differences were found, were those with mild-major (instead of a minor) depression, as diagnosed by the PCP. Patients receiving UCandAD who were diagnosed with a mild-major depression had overall lower mean MADRS scores in the per-protocol analysis (mean difference -2.9 in the per-protocol analysis, versus 1.2 in the intention-to-treat analysis).

### Patient's treatment preferences

Explorative analyses showed that patient's treatment preferences were related to MADRS scores at 13 weeks and 26 weeks follow-up in the intention-to-treat analysis. At 13 weeks, patients who preferred UCandAD or UCnoAD and received their preferred treatment had lower MADRS scores. When the patient's preference for UCnoAD was in line with the PCP's preference and with treatment assignment, the positive effect increased (mean UCandAD 15.3; SD 13.9 and UCnoAD 12.1; SD 7.7 vs overall mean UCandAD 13.7; SD 11.1 and mean UCnoAD 15.7; SD 12.3). At 26 weeks, patients who preferred and received UCnoAD had lower MADRS scores (mean UCandAD 11.0; SD 11.4 and UCnoAD 10.2; SD 9.4 vs overall mean UCandAD 11.3; SD 10.7 and mean UCnoAD 14.0; SD 11.8). No relation between patients' treatment preferences and MADRS scores was found in the per-protocol analysis.

### Secondary outcomes

#### SF-36

No significant differences on the PCS scale or the MCS scale (SF-36) between the treatment groups were found at any time point. For example, the mean score on the PCS scale at 13 weeks follow-up was 43.8 (SD = 8.0) in UCandAD and 46.1 (SD = 7.9) in UCnoAD. The mean score on the MCS scale at 13 weeks follow-up was 40.5 (SD = 11.0) in UCandAD and 37.8 (SD = 11.6) in UCnoAD.

#### CSQ-8

At 13 weeks follow-up, but not at 52 weeks, patients allocated to UCandAD were modestly more satisfied with their treatment than patients allocated to usual care alone (mean item difference 0.18; p = 0.04).

## Discussion

We conducted a large, pragmatic randomized equivalence trial with 52 weeks follow-up in primary care patients with minor and mild-major depression. We compared usual care with antidepressants (UCandAD) to usual care without antidepressants (UCnoAD) and hypothesized that they were equally effective, i.e. equivalent. The results showed that equivalence of UCandAD and UCnoAD was demonstrated after 6 weeks of treatment in both the intention-to-treat analysis and the per-protocol analysis. Equivalence could not be demonstrated from 13 weeks on. Therefore, one of the two treatments could be more effective from 13 weeks on. We took the next step and explored whether UCandAD was more effective than UCnoAD. No statistical significant differences were found between the treatments in the intention-to-treat analysis. In summary, from 13 weeks on, there is no hard evidence that both treatments are equally effective, but neither that antidepressant medication adds substantial effectiveness to usual care alone.

No differences between treatment groups in the physical and mental functioning were found. Patients allocated to UCandAD were more satisfied with their treatment in the short run (at 13 weeks follow-up) than patients allocated to UCnoAD. However, differences were small.

Are there no indications that antidepressants might have some additional effectiveness over usual care alone? Figure 2, which visualizes the results of Table 2, suggests that there are on average small differences between the treatments in favour of UCandAD. In the per-protocol analysis statistical significant differences between the treatment groups were found at 26 and 52 weeks follow-up. Despite the initial lack of benefit of antidepressant medication, the differences increased over time and were maintained until the last follow-up assessments at 52 weeks, long after the treatment had ended. Therefore, it is not very likely that this effect was purely due to a placebo effect. These outcomes should be weighted carefully, as they differ from the results of the intention-to-treat analysis and protocol violation more often occurred in UCandAD. However, given the heterogeneity of depressive disorders, some subgroups of patients may actually benefit from antidepressants. We found an indication that in particular patients with mild-major (instead of minor) depression may benefit from additional antidepressant medication.

A recent review on the treatment of depression in primary care concluded that patients with strong preferences, in particular when psychotherapy is preferred, are likely not to enter randomized clinical trials if their preferences are not supported [30]. We also found some support for this phenomenon. Because of the randomization procedure, 42 patients choose not to enter our trial. Furthermore, we found that protocol violation more often occurred in UCandAD. Patients who were not assigned to the treatment of their preference were less compliant. This was found in particular for patients who preferred UCnoAD or had no treatment preference, but were assigned to UCandAD. The review also concluded, based on results of partially randomized patient-preference trials, that there is no evidence that patient's treatment preference predicts clinical outcome. However, our results indicate that patient's treatment preferences were positively related to clinical outcome, again in particular for patients who preferred and were assigned to UCnoAD.

Our study had some limitations. The combination of a pragmatic and an equivalence trial turned out to be difficult. Comparison of the unadjusted results in Table 2 and the adjusted results in Table 3 show that co-interventions have diluted differences between treatment groups. Moreover, more than 25% of the patients were not treated according to the protocol (Figure 1). Protocol violations reduce the contrast between the treatments. This is considered a problem in equivalence trials, because it increases the risk of erroneously concluding equivalence (type I error) [29,31]. However, in pragmatic trials, as in everyday practice, protocol violations are common practice. Protocol violations therefore determine the effectiveness of an intervention under normal circumstances.

There are very few controlled trials in primary care examining the effectiveness of antidepressant treatments for minor and major depression, and these were designed to prove superiority of antidepressants over placebo or problem solving treatment [32-36]. None of these studies could prove superiority of antidepressant medication. These results raise questions how liberal physicians should be in prescribing antidepressants to patients in primary care [37]. Our study is the first equivalence trial on the treatment of depression in primary care, while at the same time the follow-up in our trial was much longer than in most antidepressant studies in primary care. Added to the fact that it was a pragmatic trial, mirroring normal clinical practice, the generalizability is high.

## Conclusion

Usual care combined with paroxetine (UCandAD) was as effective as (equivalent to) usual care alone (UCnoAD) over the first 6 weeks, but not thereafter. We found small differences in effectiveness in favor of UCandAD, but superiority was not demonstrated. We cannot but conclude that the question 'Do antidepressants add any effectiveness to usual care?' remains open. We recommend future studies to distinguish between subgroups of patients who might benefit from antidepressant medication. Until then, the potential benefits of adding an antidepressant to usual care alone must be balanced judiciously against possible harms such as side effects and dependence. Moreover, patients should be properly informed about the advantages and disadvantages of interventions in order to enable them to make a balanced choice.

## Competing interests

The author(s) declare that they have no competing interests.

## Authors' contributions

MH, HvH, BT, RvD, and MdH contributed to the design of the study and coordinated the conduct of the trial and the data collection. All authors made substantial contributions to the design of the manuscript. MH and HA performed the statistical analyses; HvH and BT contributed to the interpretation of the data. MH drafted the manuscript and the other authors revised it critically. All authors read and approved the final version of the manuscript.

## Pre-publication history

The pre-publication history for this paper can be accessed here:


